# The Use of Single Cell Mass Cytometry to Define the Molecular Mechanisms of Varicella-Zoster Virus Lymphotropism

**DOI:** 10.3389/fmicb.2020.01224

**Published:** 2020-06-26

**Authors:** Nandini Sen, Gourab Mukherjee, Ann M. Arvin

**Affiliations:** ^1^Department of Pediatrics, Stanford University, Stanford, CA, United States; ^2^Department of Data Sciences and Operations, University of Southern California, Los Angeles, CA, United States; ^3^Department of Microbiology and Immunology, Stanford University, Stanford, CA, United States

**Keywords:** varicella zoster virus remodeling of T cells, tonsil T cell immunophenotyping, T cell signaling, single cell analyses, varicella zoster virus lymphotropism

## Abstract

Unraveling the heterogeneity in biological systems provides the key to understanding of the fundamental dynamics that regulate host pathogen relationships at the single cell level. While most studies have determined virus-host cell interactions using cultured cells in bulk, recent advances in deep protein profiling from single cells enable the understanding of the dynamic response equilibrium of single cells even within the same cell types. Mass cytometry allows the simultaneous detection of multiple proteins in single cells, which helps to evaluate alterations in multiple signaling networks that work in tandem in deciding the response of a cell to the presence of a pathogen or other stimulus. In applying this technique to studying varicella zoster virus (VZV), it was possible to better understand the molecular basis for lymphotropism of the virus and how virus-induced effects on T cells promoted skin tropism. While the ability of VZV to manifest itself in the skin is well established, how the virus is transported to the skin and causes the characteristic VZV skin lesions was not well elucidated. Through mass cytometry analysis of VZV-infected tonsil T cells, we were able to observe that VZV unleashes a “remodeling” program in the infected T cells that not only makes these T cells more skin tropic but also at the same time induces changes that make these T cells unlikely to respond to immune stimulation during the journey to the skin.

## Introduction

**Figure 1:**
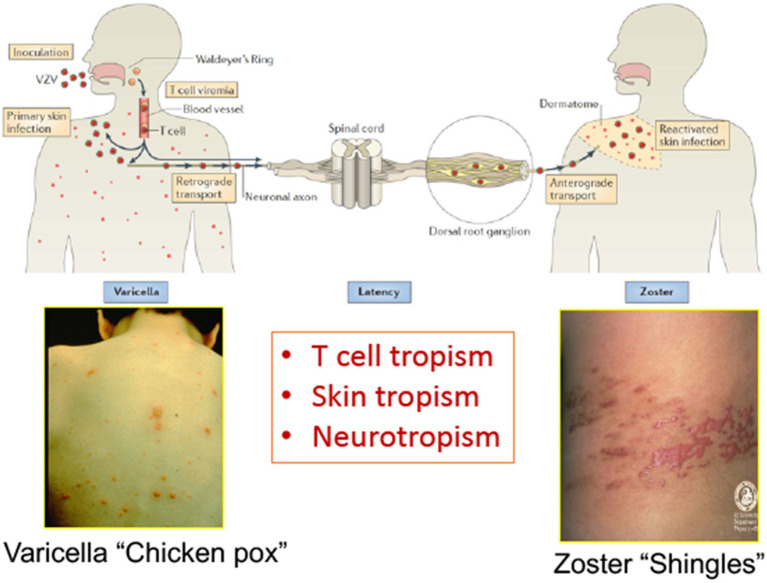
The life cycle of varicella zoster virus (VZV) in the human host. VZV infects the human host when virus particles reach mucosal epithelial sites of entry. Local replication is followed by spread to tonsils and other regional lymphoid tissues, where VZV gains access to T cells. Infected T cells then deliver the virus to cutaneous sites of replication. VZV establishes latency in sensory ganglia after transport to neuronal nuclei along neuronal axons or by viremia. Reactivation from latency enables a second phase of replication to occur in the skin, which typically causes lesions in the dermatome that is innervated by the affected sensory ganglion. Adapted from Zerboni et al. (2014).

Varicella zoster virus (VZV) is a medically important human alphaherpesvirus that causes varicella (chickenpox) as the manifestation of primary infection and herpes zoster (shingles) when it reactivates from latency (Figure 1; Zerboni et al., 2014). Both primary and recurrent VZV infections result in skin lesions, and like other alphaherpesviruses; VZV persists in neurons within sensory ganglia. Recent investigations of VZV neurotropism have used neural progenitor-derived tissue assemblies (Sadaoka et al., 2016) and neuronal stem cells to model persistence and reactivation (Markus et al., 2015). Axonal transport leads to infection of peripherin+ nociceptive and RT97+ mechanoreceptive neurons whereas RT97+ neurons do not become infected; VZV spreads between neurons and satellite cells in sensory ganglia (Zerboni and Arvin, 2015).

**Figure 2:**
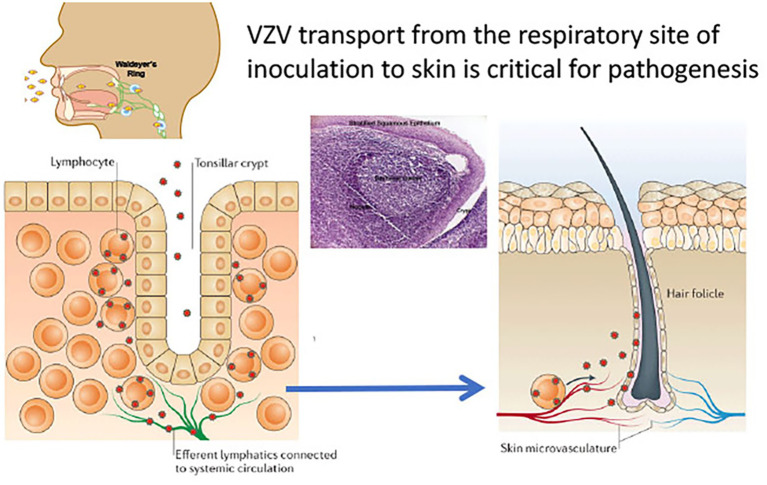
VZV tropism for tonsil T cells. According to the model of VZV cell associated viremia, tonsil T cells are infected following VZV inoculation and replication in respiratory mucosal epithelial cells. T cells traffic into and out of tonsils across the squamous epithelial cells that line the tonsillar crypts (left panel). Single cell high dimensional multiparametric studies showed that VZV infection of the tonsil T cells induces remodeling that alters the T cells to a phenotype that promotes skin trafficking. Adapted from Zerboni et al. (2014).

However, VZV is distinguished from the other alphaherpesviruses by the capacity to infect T cells in addition to its tropisms for skin and neurons. The life cycle of VZV in the human host is thought to begin with inoculation of mucosal epithelial cells of the oropharynx, where the virus can gain access to the T cells within tonsils or other regional lymphoid tissues that make up Waldyer’s ring (Figure 2; Zerboni et al., 2014). The lymphotropism of VZV is essential for the pathogenesis of primary infection as well as for the establishment of latency in sensory ganglion neurons since VZV is delivered to skin sites of replication by T cells during primary infection and to sensory ganglia either by retrograde transport along neuronal axons from varicella skin lesions or directly to ganglia by VZV-infected T cells (Ku et al., 2002; Schaap et al., 2005; Zerboni et al., 2005). VZV is maintained in the human population by transmission of infectious virus in aerosolized droplets from infection of respiratory epithelial cells or by contact with the varicella or zoster skin lesions that contain high concentrations of infectious virus (Zerboni et al., 2014).

Having demonstrated that tonsil T cells are readily infected by VZV and release infectious virions, we proved that the transfer of VZV into human skin was a biologically functional mechanism for VZV pathogenesis *in vivo*. When VZV-infected tonsil T cells were injected into the circulation of immunodeficient severe combined immunodeficiency disease (SCID) mice that had human skin xenografts, T cells exited into skin tissue and typical VZV skin lesions were formed, whereas other types of human cells did not transport VZV to the skin *in vivo* (Ku et al., 2004). In the skin, we have observed that the virus encounters a potent innate defense barrier mediated by the Type I IFN response, which correlates with the long (10–21) day incubation period before primary VZV infection results in the typical cutaneous rash. Analyses of infected skin xenografts suggest that after infected T cells exit into the skin, VZV infects cells at the base of the hair follicles, which are predominantly epithelial stem cells, and triggers several signaling changes that function to block innate immune responses. For example, phosphorylation of STAT3, which upregulates survivin expression, was found to be required for VZV infection of skin (Sen et al., 2012). That VZV infected tonsil T cells can also transport the virus to sensory ganglia was shown in SCID mice with human dorsal root ganglia xenografts (Zerboni et al., 2005). Therefore, deep profiling the underlying proteomic nature of VZV lymphotropism is important not only for VZV pathogenesis but is also important because infection of immune T cells is responsible for much of the morbidity associated with VZV, including dissemination to liver and lungs in immunocompromised patients and transplacental transfer with the risk of intrauterine infection of the fetus and varicella pneumonia in adults. In addition, while the vaccine strain of VZV is restricted for growth in skin, its ability to successfully infect T cells preserves the possibility of an infection from vaccine in immunocompromised individuals (Moffat et al., 1995).

Here, we review our work using single cell mass spectrometry to show that the transportation of VZV by T cells to skin occurs through an active remodeling process, whereby the virus modulates host cell signaling pathways to promote the preferential trafficking of infected tonsil T cells to the skin. We also provide new analyses of the initial single cell data set that provide further insights about the molecular mechanisms of VZV lymphotropism.

## Rationale for Investigating VZV Tropism for Differentiated Host Cells Using a Single-Cell Approach

In designing experiments that would elucidate VZV tropism for human tonsil T cells, we considered the limitations of the usual approaches for studies of interactions between virus and host cell proteins. For the most part, the consequences of viral replication are determined in cells or cell lines considered to have characteristics resembling target cells that are involved in viral pathogenesis and are then infected with the virus of interest and evaluated as bulk cultures. There is no doubt that investigating the functions of specific viral proteins and changes in expression of the cell proteins that are triggered by viral infection in a uniform population of cultured cells can provide important insights about the effects that are identifiable by averaged measurements. However, *in vivo*, viral pathogens encounter differentiated host cells exhibiting their inherently stochastic states and the process of altering these conditions to support pathogenesis must occur within each cell. Methods that rely on cells in bulk culture do not offer a comprehensive assessment of the induced molecular changes when the virus infects differentiated primary cells from the native host. One issue is the heterogeneity of cell signaling at baseline even when the bulk cultures consist of the same cell line. Signaling changes associated with phosphorylation and other protein modifications are often transient in nature and perturbations due to viral infection are difficult to capture when the changes are occurring in only a fraction of cells. Another challenge is achieving synchronous infection, which even if obtained, typically requires high multiplicities of infection that are often not physiologically representative of conditions during viral pathogenesis *in vivo*.

In contrast, single cell mass cytometry offers the opportunity to measure multiple concurrent changes in protein expression in individual cells, which is necessary for documenting how viruses manipulate differentiated primary host cells that are characterized by heterogenous signaling states (Bendall et al., 2011; Spitzer and Nolan, 2016). As we review here, our work on VZV T cell tropism substantiates major advantages of this approach as follows: (a) testing single cells revealed that VZV orchestrates a continuum of changes within heterogeneous host cells, regardless of their basal state, which could not be shown by averaged measurements and (b) multi-parametric analysis of single cells was vital because there is no one “skin homing” marker on human T cells which could be measured to prove this functional consequence of VZV infection. A constellation of changes is needed, including both upregulation and downregulation of T cell surface proteins to elicit a skin trafficking profile, and (c) simultaneous measurements of multiple phosphoproteins made it possible to show that VZV exploits known T cell pathways typically triggered through the T cell receptor (TCR) and to link surface changes to concurrent VZV activation of cell signaling proteins. In summary, we found that single cell mass cytometry accompanied by rigorous statistical analysis is a robust new tool to study virus-mediated manipulation of primary host cells by making possible multi-parametric measurements from more than 50 million single T cells. As illustrated by our experiments, viruses can mimic perturbations mediated by host cytokines to alter cell surface properties of infected cells, while at the same time orchestrating changes to avoid immune recognition and clearance.

## The Application of Single Cell Mass Cytometry to Investigate VZV T Cell Tropism

The technique of single cell mass cytometry, termed CyTOF, uses metal isotope labeled antibodies to detect protein expression profiles for individual cells (Bendall et al., 2011). Use of metal isotope labeling allows the simultaneous detection of many more individual proteins than is feasible with fluorescent labeled antibodies. Several discovery studies using mass cytometry have been reported since 2011, including identification of rare transient cell clusters in disease conditions and our study of VZV T cell infection, which was the initial report showing the value of this technique for investigating virus perturbations of differentiated host cells, followed by its application to T cell infection by HIV (Cavrois et al., 2017).

**Figure 3:**
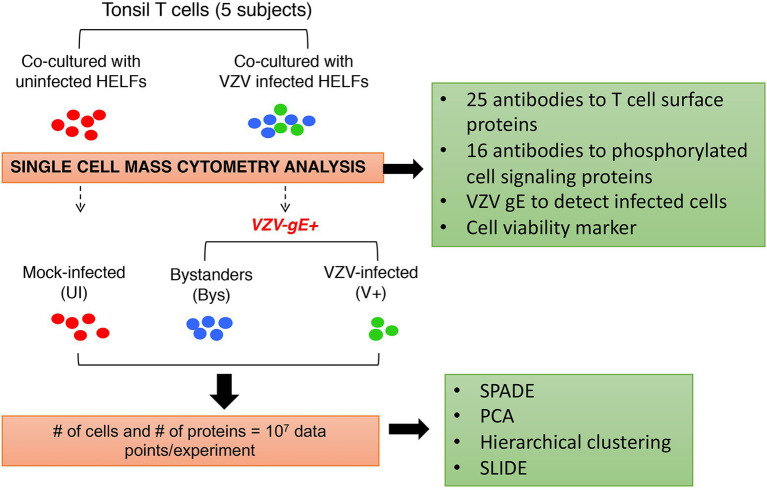
Experimental approach for analysis of VZV infection of T cells at the single cell level. Primary human tonsil T cells were isolated from tonsils collected from patients undergoing tonsillectomy. The T cells were purified from other immune cell types by negative selection and were exposed to virus infection by co-culturing with infected primary fibroblasts [human embryonic lung fibroblasts (HELFs)]. T cells were also co-cultured with mock-infected fibroblasts (mock infection) to control changes in T cells associated with co-culturing. The mock‐ and VZV-infected T cells were collected, fixed at 48 hpi, and stained using a cocktail of antibodies against a predefined set of host cell surface and intracellular signaling targets. T cells containing virus were identified by glycoprotein E (gE) expression (green) and distinguished from non-gE expressing “bystander” cells (blue), which were exposed to infected fibroblasts but did not become infected by VZV. For all downstream analysis, the VZV-infected (gE expressing) cells were compared to the “bystander” and mock-infected cells, both of which were controls for recording changes, specifically introduced by VZV. Several analytical algorithms including spanning-tree progression analysis of density-normalized events (SPADE), principal component analysis (PCA), hierarchical clustering, and single cell linkage using distance estimation (SLIDE) were applied in this study. Adapted from Sen et al. (2014).

In our studies of VZV pathogenesis, single cell mass cytometry made it possible to overcome major challenges in the investigation of VZV T cell tropism, including that tonsil T cells are heterogeneous differentiated primary cells, signaling pathways in these cells are in varying states of activation and deactivation, and transformed T cell lines do not reproduce the diversity of primary T cells encountered by the virus in the natural host. Our strategy was designed to document VZV effects on the functional capacities of differentiated primary T cells by investigating combinatorial changes in T cell proteins with known functions, the cell surface expression of 25 T cell proteins associated with T cell activation and skin homing and the phosphorylation state of 16 proteins involved in intracellular signaling in T cells. The experimental workflow was as illustrated in Figure 3. Primary human tonsil T cells were isolated from tonsils collected from patients undergoing tonsillectomy. The T cells were purified from other immune cell types by negative selection and were exposed to virus infection by co-culturing with infected primary fibroblasts [human embryonic lung fibroblasts (HELFs)]. T cells were also co-cultured with mock-infected fibroblasts (mock infection) to control changes in T cells associated with co-culturing. T cells were collected, fixed at 48 hpi, and stained using a cocktail of antibodies for downstream CyTOF analysis. T cells containing virus were identified by glycoprotein E (gE) expression and distinguished from non-gE expressing “bystander” cells, which were exposed to infected fibroblasts but did not become infected by VZV. Cells analyzed for changes in protein expression were chosen following a sequential gating procedure to identify intact viable cells. Here, we provide an overview of observations about VZV infection of T cells obtained by single cell mass cytometry.

## Immunophenotyping

**Figure 4:**
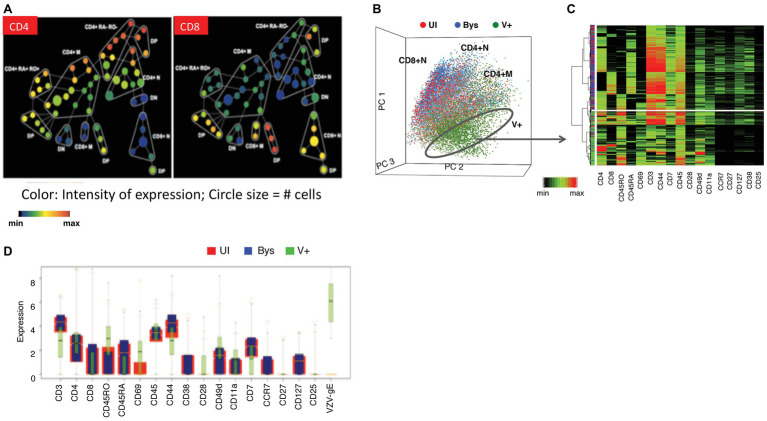
Establishing the changes in the phenotypic hierarchy of mock-infected tonsil T cells following VZV infection. **(A)** SPADE tree of mock-infected T cells. The mock-infected, bystander and infected T cells were distributed by SPADE analysis tool (www.cytobank.org) into 50 nodes, each node representing a collection of cells with similar expressions of four core markers – CD4, CD8, CD45RO, and CD45RA. The size of each node correlated with the proportion of cells within each node and the color intensity is proportional to the expression of the indicated proteins; left panel shows the distribution of CD4 expression while the right panel shows that of CD8. Tonsil T cell subpopulations were highly consistent among subjects. **(B)** PCA of mock-infected, bystander, and VZV-infected T cells shows the distribution of the cells (each dot is a single cell) in 17-dimensional space determined by the expression of 17 surface markers evaluated in this analysis. The mock-infected cells (red dots) and bystander cells (blue dots) showed a neat overlap being distributed into three main cell clouds consistent with CD4^+^ memory, CD4^+^ naïve, and CD8^+^ naïve cells. The VZV-infected cells (green dots) on the other hand occupied a distinct niche on the PCA plot. **(C)** Agglomerative hierarchical clustering of the cells, shown as a heatmap, clusters the VZV-infected cells distinctly from the mock-infected and bystander cells, which show overlapping distribution. **(D)** The box plots show the differential expression of the individual surface proteins between the mock-infected (red) and VZV-infected (green) cells. Adapted from Sen et al. (2014).

As background for studies of VZV infection of T cells, we first established the phenotypic hierarchy of mock-infected tonsil T cells (Figures 4A,B). The scatter plot of the principal component analysis (PCA) of the mock-infected tonsil T cells distributed them into three broad clusters, including the CD4^+^ memory and naïve cells and CD8^+^ naïve cells. Spanning-tree progression analysis of density-normalized events (SPADE) analysis subgrouped the cells further into cell clusters with differential expression of CD4, CD8, CD45RO (memory), and CD45RA (naïve) markers, including cell clusters that were dual positive for CD45RO/RA and/or negative for both these proteins. Interestingly, the bystander cells, that were co-cultured with the infected HELFs but did not become infected, showed an identical PCA and SPADE profile when compared to the uninfected control T cells.

Next, we evaluated the effects of VZV on the phenotypic hierarchy of tonsil T cells (Figure 4B). On the PCA scatterplot, the infected cells occupied a space that was distinct from both the mock-infected or bystander T cells. An agglomerative hierarchical clustering analysis of the mock-infected, bystander, and infected cells, shown as a heatmap (Figure 4B), distinguished the virus infected cells on a distinct dendrogram. This unique clustering of the infected cells was a consequence of altered expression of several T cell surface proteins, as illustrated in Figures 4C,D. VZV infection altered the expression of T cell surface proteins within and across T cell subpopulations, including naïve and memory T cells. The functional implications of the T cell surface changes were that changes of T cell surface proteins on VZV-infected T cells reflected activation and changes leading to an effector phenotype which supports tissue homing, including increased CD69, PD-1, and CD45RO. Enhanced skin homing potential was also reflected by increased expression of integrins, CD11a and CD28, along with enhanced CCR4 and CLA and decreased expression of CCR7 and CD7. Changes associated with impaired T cell immune function included increased PD-1 and reduced CD3ε, which would be likely to interfere with any immunological activity of infected T cells during viremia. Notably, surface expression of proteins on bystander T cells was unchanged compared to mock-infected controls despite exposure to VZV-infected fibroblasts and T cells in the cultures. These observations indicate that changes are independent of cytokine effects known to activate T cells and alter cell surface proteins during an antigen-specific T cell response and most importantly, that altered cell surface proteins resulted from changes induced from within infected T cells.

**Figure 5:**
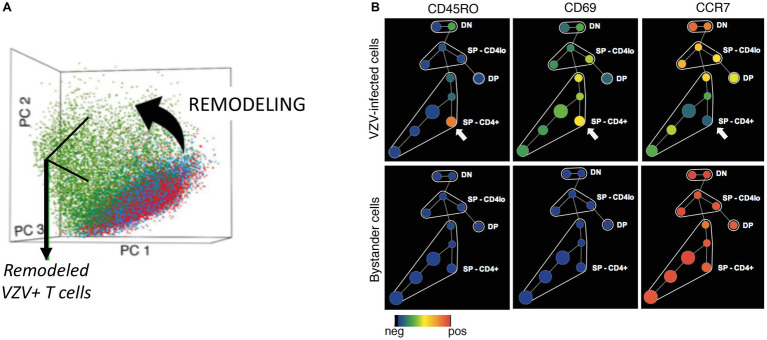
Verification of the remodeling capacity of VZV infection demonstrated in naïve T cells. **(A)** PCA analysis of CD4^+^ naïve mock-infected, bystander, and VZV-infected T cells is shown as a scatterplot, where a dot represents a single cell. The mock-infected (red dots) and bystander (blue dots) cells occupied a single cell cloud based on CD4, CD8, CD45RO, and CD45RA expression. In contrast, the VZV-infected cells (green dots) had a unique distribution on the plot reflecting the “state transition” or remodeling of the naïve T cells to express phenotypes that marked them for skin trafficking. **(B)** SPADE analysis for visualization of the differential expression of surface proteins between VZV-infected and bystander cells. Adapted from Sen et al. (2014).

In order to confirm these effects of VZV on T cell surface protein expression, the remodeling capacity of the virus was experimentally examined using purified naïve T cells, in the absence of other T cell populations that were co-cultured as described above. The mock‐ and VZV-infected naïve T cells were similarly assessed for changes in surface and signaling proteins (Figure 3). In Figure 5A, PCA scatterplot shows that the effects of VZV-infection on the naïve T cells were distributed distinctly from the mock-infected and bystander cells (red and blue dots). The more scattered distribution of the VZV-infected T cells (green dots) showed that VZV induced remodeling directs alterations, resulting in a widely heterogenous population of T cells; the heterogeneity is most likely contributed by the fact that these cells are at different stages of virus infection/replication. This remodeling was a direct consequence of VZV induced changes in expression of several surface proteins of the naïve T cells to impart an activated, skin tropic effector phenotype without which, naïve T cells would not be expected to leave the tonsils and traffic to the skin (Figure 5B).

## Quantitative Analysis of Immunophenotypic Alterations

Based on these findings, a combinatorial analysis of the expression of T cell surface proteins was carried out to address the critical question about whether VZV infects cells that are pre-programmed with the observed attributes, which would represent preferential infection or whether VZV infection reconfigures the surface of T cells to produce the profiles that were observed, which would constitute remodeling. To address this question, a combinatorial analysis of the 13 surface proteins on mock-infected and infected T cells within the subpopulations identified by the CD4, CD8, CD45RA, and CD45RO core markers was done using an analysis that we termed single cell linkage using distance estimation (SLIDE; Sen et al., 2014). SLIDE is a statistical test based on the nearest neighbor method. There has been an explosion of analytical tools for CyTOF data analysis most of which are used for determining patterns and progression of changes in cells and tissues isolated from patients. While methods like SPADE, PCA, and agglomerative clustering analysis allowed the depiction of virus infected cells as a distinct population in multidimensional space, SLIDE analysis helped us to quantify the remodeling capacity of the virus at single cell levels (Sen et al., 2015). Therefore, data analysis by each these methods enabled a complementary and robust approach to multidimensional single cell data analysis.

## Remodeling

**Figure 6:**
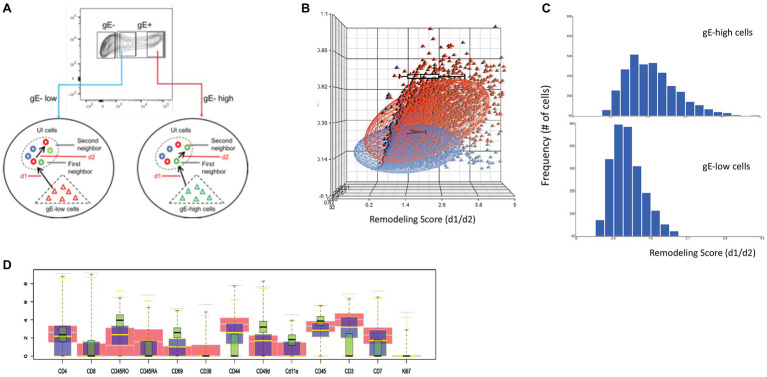
Single cell linkage using distance estimation. **(A)** Schematic representation of the two-step nearest neighbor analysis for single cell linkage distance estimation (SLIDE) in gE-low and gE-high expressing VZV-infected cells. Each VZV-infected T cell was matched with every mock-infected T cell to identify its closest neighbor based on combinatorial expression of 13 non-core surface markers and the absolute distance (d) between the two cells were recorded as d1. The nearest neighbor mock-infected T cell identified by d1 was subsequently matched to a second nearest neighbor in the same mock-infected population and the distance between the two mock-infected T cells was measured as d2. The remodeling score was estimated by calculating the d1:d2 ratio. **(B)** Three-dimensional scatterplot of d1, d2, and d1:d2 ratio for each VZV-infected cell. The blue dots are cells that express low gE while the red dots are those that express high-gE. The remodeling score (d1:d2 ratio) is shown in the x-axis; d1 is plotted on y axis while d2 is plotted on z-axis. **(C)** Histogram analysis showing the frequency of cells for the spectrum of remodeling score calculated for the gE-low and gE-high cells. **(D)** The box plots show the differential expression of the individual surface proteins between the mock-infected (red) and VZV-infected cells expressing low (blue)‐ and high (green)-gE.

In our previous remodeling analysis using SLIDE, we showed that within each of the major T cell subsets, the infected cells underwent phenotypic changes (remodeling) induced by the virus. For this review, we further delved into the remodeling analysis by SLIDE to determine if there was a correlation between the remodeling of the infected cells and levels of VZV-gE expression, used as a marker of the extent of VZV replication. For that purpose, we categorized the infected cells from each experiment into two groups: (a) 30% cells expressing high gE and (b) 30% cells expressing low GFP (Figure 6A). A total of approximately 14, 000 single infected cells, expressing high and low gE, were analyzed by SLIDE. The SLIDE ratio, which quantifies the distance between infected and nearest neighbor mock-infected cells, increased with gE expression indicating increased remodeling of these cells in the presence of replicating virus. The scatterplot, shown in Figure 6B, shows the distribution of the remodeling score of individual infected cells (dots) expressing low (blue) and high (red) gE; compared to the low gE expressing cells, the gE-high cells showed higher d1/d2 ratio (*x*-axis). To evaluate the significance of the difference in the SLIDE ratio of the gE-low and gE-high cells, we used effect size, a statistical measure used to quantify an observed phenomenon. The magnitude of difference between the two populations was >2.0, indicating a significant change in SLIDE ratio between the two groups of cells that were in earlier or later stages of infection. The frequency of gE-high and gE-low cells (*y*-axis) with their corresponding remodeling score (*x*-axis) was plotted as a comparative histogram to show the distribution of the degree of remodeling in the infected cells (Figure 6C).

The remodeling of infected cells was calculated based on simultaneous bidirectional changes in surface expression of T cell markers that were assessed in the panel. The box plot in Figure 6D shows the differences in expression of the indicated individual surface markers in the gE-high (green) and gE-low (blue) populations compared to the mock-infected cells (red). The above analysis showed a direct correlation between virus replications, determined by the expression of VZV-gE and the extent of remodeling in the infected cells.

## Intracellular Phosphoproteomic Alterations

**Figure 7:**
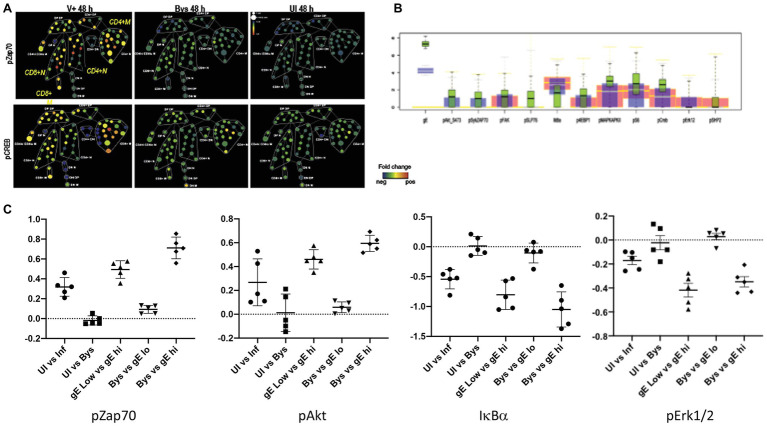
VZV induces signaling through the T cell receptor (TCR) pathway. **(A)** SPADE tree showing the fold-change in expression levels of pZap70 and pCREB in VZV-infected and bystander T cells relative to mock-infected T cells by SPADE analysis. In this case, the node colors indicate fold-change in expression in phosphorylated proteins, from minimum (blue) to maximum (red, relative to the mock-infected cells). **(B)** The box plots show the differential expression of the individual phosphoproteins between the mock-infected (red) and VZV-infected low-gE expressing (blue) and high-gE expressing (red) cells. **(C)** Effect size of the indicated proteins when compared between the indicated groups of cells (x-axis), where the data plotted on y-axis is of the second group compared to the first group.

The capacity of single cell mass cytometry to detect the phosphorylation state of intracellular signaling proteins made it possible to address the next critical question, which was how VZV infection results in the remodeling of T cell surface proteins. Using this approach permitted the documentation of VZV modulation of intracellular T cell signaling. The context for including the phosphoprotein analysis was that the expression of T cell surface proteins is known to be regulated by intracellular signaling pathways to achieve T cell differentiation and support immune functions and that high dimensional quantitative single cell measurements can capture the inherently stochastic nature of signaling. When T cells respond to cognate antigen in mounting a typical immune response, T cell receptors activate a signaling cascade *via* the TCR-Zap70 and TCR/CD28-FAK-Akt pathways. Since VZV induced a combination of cell surface changes, we asked whether the cell surface changes on VZV-infected T cells were associated with activation of the typical intracellular signaling cascade triggered by the response to a cognate antigen. As with surface antigens, analysis of the CyTOF data to measure signaling changes also involved various algorithms including SPADE (Figure 7A). Rigorous conventional statistical tests were also applied to evaluate the changes which were more subtle (compared to the surface marker changes) given the transient nature of activation of the proteins involved in cell signaling pathways (Figures 7B,C). The boxplots show that the gradient change in expression of the phosphoproteins correlated with increasing gE expression. In addition, the effect size for each of these phosphoproteins was calculated between several comparative groups (Figure 7C). An effect size of >0.2 indicates significant changes in expression of the phosphoproteins. Of note, the effect size of the bystanders versus mock-infected cells remained at <0.1, indicating that signaling pathways were unchanged in the bystander cells. This observation using effect size not only confirmed our previous findings but also provided a quantification of the magnitude of observed changes between different groups.

The CyTOF analysis of infected T cells showed that VZV-induced effects on both of the well-known T cell signaling pathways played a major role in T cell remodeling. Our findings revealed that both pathways were activated in infected cells and elucidated by phosphorylation of several key pathway proteins and that the activation intensity was proportional to the expression of gE in the infected cells. VZV infection triggered pathways that are observed when T cells respond to an antigen, which are mediated by TCR stimulation *via* the major histocompatibility complex in the case of Zap70 and for Akt signaling, by ligand binding to co-stimulatory molecules, e.g., CD28. Phosphorylation of multiple downstream proteins indicated functional activation of the intracellular signaling pathway. Notably, activation was documented in memory and naïve T cells, but was more pronounced in memory T cells. However, the occurrence of activation in naïve T cells even when basal conditions are most challenging to induce signaling demonstrated the potency of the VZV-induced takeover of these pathways. The absence of activation of both these pathways in the bystander cells demonstrated that the modulation of cell signaling was due to VZV activity triggered from within the infected T cells.

Taken together, these studies showed that VZV infection stimulates signaling at the early stages of the mechanisms which underlie the plasticity of T cells required for immune responses to a cognate antigen. VZV modulation occurred in the context of the basal state, which varies cell to cell. Not surprisingly, effects were more pronounced in memory T cells, which have a lower activation threshold. The importance of the multi-parametric assessment made possible by CyTOF was illustrated by our finding that the effects of VZV infection were selective in that downstream protein phosphorylation can be blocked or reversed. For example, Erk1/2, which is important for TCR-regulated production of the antiviral cytokines that would be expected to inhibit replication was blocked in VZV infected T cells. A fundamental insight using this experimental approach was that the induction of T cell signaling by VZV infection is T cell receptor and cytokine independent and occurs from within. Further, the signaling changes detected were consistent with the altered T cell surface architecture, showing that VZV remodels T cells to elicit or enhance effector memory skin homing properties instead of preferentially infecting T cells that already have these properties.

## Functional Outcomes of Remodeling

To summarize, simultaneous changes in several factors accounted for remodeling of the infected T cells. The functional consequences of such changes depend on the combination of changes in the surface markers. Of note, changes in each surface marker could result in several functional outcomes, depending on changes in other markers in the same cell. The outcomes included:

- Activation: changes in proteins expressed by the infected cells correlated with T cell activation both at the level of surface and intracellular markers. Activation indicators included the upregulation of proteins like CD69 and PD-1. In addition, VZV infection triggered the typical pathways for the T cells response to antigens that bind and activate the TCR and other co-stimulatory molecules like CD28 and CD49d. Expression of both these surface proteins were enhanced in infected cells. The phosphorylation of several signaling molecules downstream of TCR and other receptors in infected cells indicated the flow of signaling information from the upstream intracellular kinases that respond to the cell surface receptor stimulation and to transcriptional factors that initiate transcriptional and proteomic changes in cells.
- Enhanced skin homing potential: the phenotypic and functional changes in infected cells favored a skin homing profile. This provides the molecular basis for how T cells could carry the virus from the oropharyngeal location to the skin where the virus manifests in the form of chicken pox during primary infection. The increased expression of integrins like CD11a and CD28 together with higher levels of expression of CCR4 and CLA provided a skin tropic phenotype. In addition, the decreased expression of CD3ε and CD7 on the infected cells correlated with observations from the T lymphoma cells that cause cytotoxic T cell lymphoma (CTCL), which also manifested in the form of skin rashes (Labastide et al., 1990).
- Impaired immune function: the expression of the immune checkpoint protein PD-1 indicated that these cells were unlikely to respond to immune surveillance function, thereby permitting successful transport of virus to the target site.

## Conclusion

Viruses are among the most common and medically significant causes of cell perturbation. As shown by our studies of VZV lymphotropism using mass cytometry, the specific consequences and the extent of virus perturbation correlate with virus replication and expression of the viral factors that regulate cell function. The significant principle about pathogenesis that emerged from this work is that a virus such as VZV, which is highly adapted to its host, could effectively reconfigure differentiated target cells to support infection despite their considerable diversity at the single cell level. Therefore, single cell mass cytometry can reveal the complex “systems biology” of virus-host cell interactions and provide a more compelling tool for identification of novel therapeutic targets in the context of changes occurring in multiple cellular networks that regulate critical cell functions.

## Author Contributions

NS performed the original laboratory experiments and data analysis, prepared manuscript, and figures. GM performed statistical analysis of the CyTOF data in the original work and new analysis done for this review. AA supervised the research, and reviewed and edited the manuscript.

### Conflict of Interest

The authors declare that the research was conducted in the absence of any commercial or financial relationships that could be construed as a potential conflict of interest.
